# Classification of Grain Amaranths Using Chromosome-Level Genome Assembly of Ramdana, *A. hypochondriacus*

**DOI:** 10.3389/fpls.2020.579529

**Published:** 2020-11-11

**Authors:** Saptarathi Deb, Suvratha Jayaprasad, Samathmika Ravi, K. Raksha Rao, Saurabh Whadgar, Nivedita Hariharan, Shubham Dixit, Meeta Sunil, Bibha Choudhary, Piergiorgio Stevanato, Eswarayya Ramireddy, Subhashini Srinivasan

**Affiliations:** ^1^Institute of Bioinformatics and Applied Biotechnology, Bengaluru, India; ^2^Department of Agronomy, Food, Natural Resources, Animals and Environment, University of Padova, viale dell’Università, Legnaro, Italy; ^3^Institute for Stem Cell Science and Regenerative Medicine (InStem), Bengaluru, India; ^4^Indian Institute of Science Education and Research, Tirupati, Tirupati, India

**Keywords:** grain amaranths plainsman, *A. hypochondriacus*, *A. cruentus*, *A. caudatus*, genotyping by sequencing, whole genome sequencing, global-germplasm resources information network (GRIN)

## Abstract

In the age of genomics-based crop improvement, a high-quality genome of a local landrace adapted to the local environmental conditions is critically important. Grain amaranths produce highly nutritional grains with a multitude of desirable properties including C4 photosynthesis highly sought-after in other crops. For improving the agronomic traits of grain amaranth and for the transfer of desirable traits to dicot crops, a reference genome of a local landrace is necessary. Toward this end, our lab had initiated sequencing the genome of *Amaranthus (A.) hypochondriacus* (A.hyp_K_white) and had reported a draft genome in 2014. We selected this landrace because it is well adapted for cultivation in India during the last century and is currently a candidate for TILLING-based crop improvement. More recently, a high-quality chromosome-level assembly of *A. hypochondriacus* (PI558499, Plainsman) was reported. Here, we report a chromosome-level assembly of A.hyp_K_white (AhKP) using low-coverage PacBio reads, contigs from the reported draft genome of A.hyp_K_white, raw HiC data and reference genome of Plainsman (A.hyp.V.2.1). The placement of A.hyp_K_white on the phylogenetic tree of grain amaranths of known accessions clearly suggests that A.hyp_K_white is genetically distal from Plainsman and is most closely related to the accession PI619259 from Nepal (Ramdana). Furthermore, the classification of another accession, Suvarna, adapted to the local environment and selected for yield and other desirable traits, is clearly *Amaranthus cruentus*. A classification based on hundreds of thousands of SNPs validated taxonomy-based classification for a majority of the accessions providing the opportunity for reclassification of a few.

## Introduction

Grain amaranth, also known as Ramdana (The God’s grain) or Rajgira or Rajeera, has been in continuous cultivation at least since last century in India. This crop was declared “The Future Crop” by the United States in the 1980s based on a decade of intense research in the 1980s (National Research Council, Amaranth: Modern Prospects for an Ancient Crop, National Academy Press, Washington, DC, 1986). At a time when gluten-free, protein-rich, high-fiber, and high nutritional values are becoming attractive labels in supermarkets around the globe, grain amaranths deserving all these labels cannot be ignored as a future crop. Furthermore, desirable agronomic traits including drought resistance, C4 photosynthesis, herbicide resistance and high dry-biomass renders grain amaranths as a potential model organism by researchers working on the improvement of other edible dicots. In the context of increasing demand on water and other natural resources from an increasing world population, grain amaranths offer an alternative to other staple cereals such as rice or wheat. With one-sixth of the world population under the poverty line, the value of the seed protein content in amaranth to India cannot be overestimated. Unfortunately, despite India being one of the few countries where multiple landraces of grain amaranths are under continuous cultivation for more than a century, they have received little attention and failed to reach the status of a staple crop.

Interestingly, grain amaranths, domesticated around 8000 years ago, enjoyed equal status as corn during the Aztec and Inca civilizations (Caselato-Sousa and Amaya-Farfán, 2012). This practice went into oblivion after the Columbian exchange. It took about 500 years after the Columbian exchange and intense efforts by the United States before this grain received the much-deserved global attention. A decade-long research conducted by the Rodale Institute during the 1980s enabled the creation of more than 800 species/varieties, which are currently maintained in a germplasm (GRIN-Global). Interestingly, this germplasm includes seeds from many amaranth landraces from South Asia including India. It is believed that these landraces, which are in contiguous cultivation in distal geographical locations in India, have already adapted to diverse environmental conditions prevalent in Nepal, as well as in East and South India.

More recently, the plummeting cost of sequencing has democratized the application of genomics technologies not only to non-model crops but has extended its reach to individual landraces with direct benefit to local farmers. In this context, the draft assembly of a landrace from India was sequenced and reported (Sunil et al., 2014). This landrace was selected for its aggressive growth, and its yield compared to a few other landraces including one with red inflorescence cultivated in India. Since then, the chromosome-level genome of a different cultivar with an accession of PI558499 (Plainsman), has been deciphered using state-of-the-art technologies including Bionano, HiC, and long PacBio reads (Lightfoot et al., 2017). This high-quality assembly has now allowed placement of about one hundred accessions from the germplasm on a phylogenetic tree (Wu and Blair, 2017) allowing for both establishing genotype-to-phenotype relationships and to place various landraces with very distinct phenotype on the tree for further characterization.

Giving chromosomal context to genes and other genetic elements is one of the most sought-after goals in genome assembly. While the genomes of hundreds of organisms at the draft stage allow deciphering the majority of the proteomes, draft genomes lack chromosomal context under which they evolve and transcribe, which is necessary for a full understanding of biology. Before long-read sequencing became commonplace, experimentally generated mate-pair reads of increasing insert sizes were routinely used to generate scaffolds from contigs. Tools, such as SOAPdenovo, use the known insert size between the mate-pair reads to connect contigs into longer scaffolds by filling the gaps with unidentified nucleotides (Ns) (Luo et al., 2012). Such an approach can simply be extended for reference-guided improvement of draft genomes of a plant using simulated mate pairs of varying insert sizes from an existing assembly of a different variety/cultivar of the same species. For example, mate-pair libraries from one *Arabidopsis thaliana* strain were shared across many strains to build super-scaffolds for all individuals (Schneeberger et al., 2011). Also, assisted assembly of closely related species significantly improved the contiguity of low coverage mammalian assemblies (Gnerre et al., 2009). The draft genomes of four species including bushbaby, African elephant, rabbit and guinea pig from the “Mammal 24 - 2X” project were built using both human and canine references (Gnerre et al., 2009). More recently, our group demonstrated that two draft genomes of the same species could be used to mutually improve scaffolding of the genome of *Anopheles stephensi* to the point that a set of low-resolution physical markers was sufficient to build the chromosomes (Chida et al., 2020).

The utility of mate-pairs from one strain to build the scaffolds for the other require DNA level similarity, which is often not the case even for closely related species. This is because DNA diverges faster even between very closely related species. However, natural selection puts sufficient selection pressure on protein sequences for maintaining functional contiguity required during evolution. In this case, one could use synteny between species at protein levels to build chromosomes. Recently, a chromosome level genome of *Lates calcarifer* was assembled from a draft genome using long-read sequencing, transcriptome data, optical/genetic mapping and synteny to two closely related seabasses (Vij et al., 2016). In yet another report, 16 out of 60 chromosomes of the Tibetan antelope were reconstructed from draft assemblies using its homology to cattle (Kim et al., 2013). In fact, using independent mapping data and conserved synteny between the cattle and human genomes, 91% of the cattle genome was placed onto 30 chromosomes (Zimin et al., 2009). In a review article, synteny has been used to filter, organize and process local similarities between genome sequences of related organisms to build a coherent global chromosomal context (Batzoglou, 2005). Similarly, the malarial strain, *Plasmodium falciparum* HB3, was improved using the reference of *P. falciparum* 3D7 combined with an assisted assembly approach that significantly improved the contiguity of the former (Gnerre et al., 2009).

Grain amaranth is yet to reach an agronomic status in India. While a large number of landraces, adapted to local environments for small scale cultivation exist, their origins and relations to the large germplasm, collection at GRIN-Global are not established. For genomics-based crop improvement of local landraces, it is critical to classify these with respect to accession from the germplasm collection. More recently, using genotyping-by-sequencing (GBS), 94 accessions for grain amaranths have been classified (Wu and Blair, 2017). While GBS is a cost-effective technology for classifying large number of accessions, it covers only 10% of the genome, which depends on the sample preparation protocol and reagents used. For a small number of landraces, it is not trivial to generate the sequences of the same 10% by reproducing the protocol/reagents, which challenges the placement of additional varieties on the phylogenetic tree generated by GBS. It is of interest to decorate this phylogenetic tree with landraces of importance to India and elsewhere.

In this article, we report a *de novo* assembly of a landrace (A.hyp_K_white) and demonstrate that, in the presence of a reference genome for a distal variety, a chromosome-level assembly can be generated at a reasonable cost. Also, we normalized the variants from GBS and WGS data for various accessions enabling decoration of the phylogenetic tree including many accessions with the landraces of interest from India.

## Results

This section is divided into three parts. The first section describes the results from our efforts to assemble a near-chromosome level assembly for a landrace A.hyp_K_white using contigs from previously reported draft genome of A.hyp_K_white (Sunil et al., 2014), low-coverage PacBio reads and a high-quality reference genome of Plainsman (Lightfoot et al., 2017). The second section describes selection and sequencing of additional landraces and varieties for classification purposes. In the third and last section, results from classification of the landraces/varieties sequenced here with sequencing data for many accessions from public resources including GBS and WGS technologies are discussed.

### Assembly of the Landrace A.hyp_K_White

**FIGURE 1:**
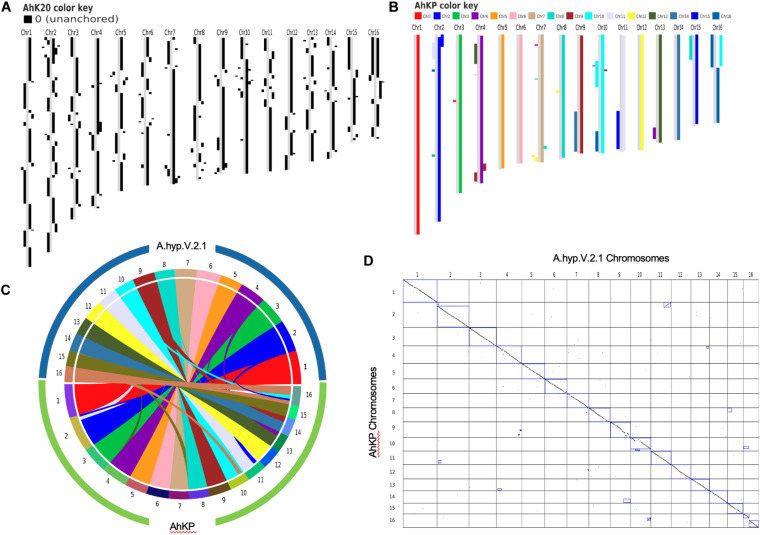
**(A)** Synteny of assemblies with respect to the Plainsman reference A.hyp.V.2.1 against AhK20. **(B)** block plot, **(C)** circular and **(D)** dot plot showing synteny of chromosomes of AhKP assembly on to Plainsman (A.hyp.V2.1).

PacBio reads using RSII technologies sequenced in 2013 with an average length of 7.5 kb with a coverage of 25X for A.hyp_K_white were assembled using state-of-the-art tools CANU (Koren et al., 2017)and FLYE (Kolmogorov et al., 2019) to obtain an assembly with L50 values of 1395 and 944, respectively, as shown in the flowchart in section “Materials and Methods” (Figure 6). These two assemblies were then merged using Quickmerge (Chakraborty et al., 2016) to improve the L50 to 623. This was further improved by merging the Illumina assembly from our previously reported draft genome of the same landrace A.hyp_K_white (Sunil et al., 2014), to get a contig-level assembly with an L50 of 593 (AhK593). This assembly was improved using HiC data from Painsman (Lightfoot et al., 2017) to obtain an assembly with a L50 of 99 (column 3, Table 1). We also improved AhK593 using simulated mate pairs from the reference genome of the Plainsman accession (Lightfoot et al., 2017), to build scaffolds of contigs from AhK593 to an L50 of 56 (AhK56) and subsequently using raw HiC data of the Plainsman accession from public sources to obtain a scaffold-level assembly with an L50 of 20 (AhK20) using SALSA (Ghurye et al., 2019). Scaffolds from AhK20 are further stitched based on synteny to A.hyp.V.2.1 (Lightfoot et al., 2017) to get the final chromosome-level assembly AhKP for the accession A.hyp_K_white. Figure 1A shows the synteny of the scaffolds from the assembly AhK20 on A.hyp.V.2.1 and Figures 1B–D show synteny of AhKP on to A.hyp.V.2.1 (Lightfoot et al., 2017) in various representations. Table 1 shows the assembly statistics.

**TABLE 1:** Assembly statistics.

**Name**	**AhK593**	**AhK99**	**AhK56**	**AhK20**	**AhKP**
**Number of contigs**	4796	2960	1926	1678	16
**Longest scaffold (Mb)**	1.83	7.00	11.41	24.01	39.67
**L50**	**593**	**99**	**56**	**20**	**7**
**N50 (Mb)**	0.19	0.89	2.00	5.40	23.02
**Assembly size (Mb)**	418.25	419.17	408.03	408.19	388.93
**Number of ATGCs**	401,504,412 (95.99%)	401,504,412 (95.78%)	366,961,434 (89.93%)	366,961,434 (89.89%)	348,890,439 (89.70%)
**Number of Ns**	1,671,100 (4.00%)	17,670,600 (4.21%)	41,071,762 (10.06%)	41,229,762 (10.10%)	40,041,419 (10.29%)

### Sequencing of Other Landraces and Ornamental Varieties

**FIGURE 2:**
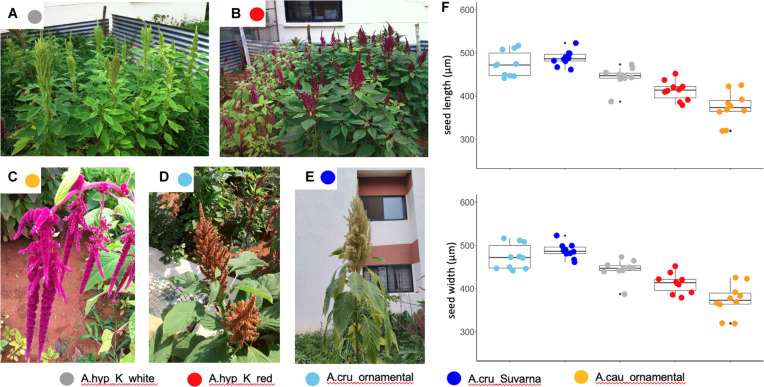
**(A)** Representative images from the institute’s field for the varieties A.hyp_K_white **(B)** A.hyp_K_red **(C)** A.cau_ornamental (love-lies-bleeding) **(D)** A.cru_ornamental (Autumn touch) **(E)** A.cru_Suvarna with white inflorescence grown at the institute campus and elsewhere for taxonomic classification **(F)** color-coded error graph of seed size for each variety.

We generated WGS data with coverage of ∼50–150X using the Illumina platform for selected landraces and ornamental varieties. Figure 2 shows the photographs of fully-grown plants sequenced and reported here. These include A.hyp_K_white (Figure 2A), A.hyp_K_red (Figure 2B), two ornamental varieties A.cau_ornamental (love-lies-bleeding, Figure 2C) and A.cru_ornamental (Autumn touch, Figure 2D) and A.cru_Suvarna (Figure 2E). A brief comparison of major agronomic traits between A.hyp_K_white and Plainsman (Supplementary Figure S1) is shown in Supplementary Table S3. The details of the sequencing are presented in Supplementary Table S1. We also downloaded WGS data from NCBI for seven other accessions including A.cau_Bolivia_PI642741, A.cru_Mexico_PI 477913, A.hyp_India_PI481125, A.hyp_Plainsman_PI558499, A.hyp_Nepal_PI619259, A.hyp_Pakistan_PI540446, A.hyp_ Mexico_PI511731, and A.hyb_Greece_PI605351. Based on the number of variants called for these and other accessions using both AhKP and A.hyp.V.2.1 as references suggest that all the landraces of *Amaranthus hypochondriacus* sequenced here are different from the A.hyp_Plainsman variety (Supplementary Table S2).

### Classification of Grain Amaranths

We have attempted to classify the landraces reported here using multiple approaches. In the first approach we have attempted to create a classification tree with WGS data for landraces sequenced here and only those accessions for which WGS data was available (NCBI Project accession SRP061623).

**FIGURE 3:**
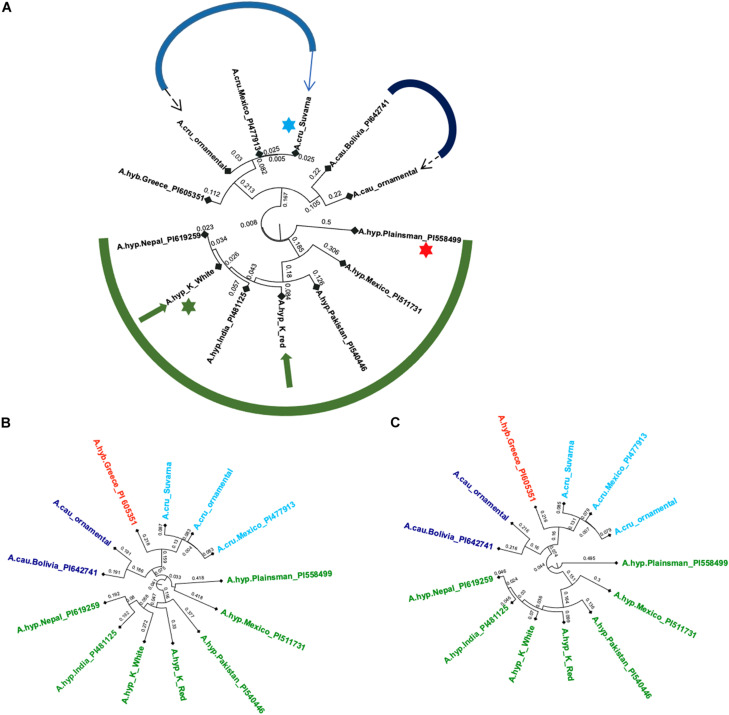
**(A)** Phylogenetic tree using the 20,548 SNPs reported for grain amaranths (green: *A. hypochondriacus*, blue: *A. caudatus*, and purple: *A. cruentus*, dashed arrows: ornamental, solid arrows: landraces with green star for A.hyp_K_white and red star for A.hyp_Plainsman variety, **(B)** Classification using 5,545,132 SNPs from the mapping of short reads onto AhKP as reference, **(C)** Classification using 6,383,490 SNPs from the mapping of short reads to A.hyp.V2.1 as reference. Light blue star points to A.cru_Suvarna.

The variants from WGS data from all the plants in Figure 2 were compared with those from the Plainsman accession and a handful of other accessions from public resources including *A. hypochondriacus, Amaranthus caudatus, Amaranthus cruentus, and Amaranthus hybridus* (Lightfoot et al., 2017). Figure 3A shows classification using the 27,658 SNPs reported for grain amaranth by Maughan et al. (2009). Of these only 20,548 positions could be found covered in all whole-genome sequencing data across all samples studied here.

All three trees in Figure 3 suggest that A.hyp_Plainsman_PI558499 (red star) is distal to the clade belonging to A.hyp_K_white (green star). The tree generated using the 20,548 SNPs (Figure 3B) is independent of any reference and hence, can be considered unbiased. On the other hand, the phylogenetic trees shown in Figures 3B,C, generated using ∼6 million SNP variants, which shows some bias from the references used to generate variants, also clusters A.hyp_K_white in a distal clade from A.hyp_Plainsman_PI558499. Also, A.hyp_K_white is closest to A.hyp_Nepal_PI619259 and A.hyp_India_PI481125 with A.hyp_K_red relatively distal from A.hyp_K_white. It is interesting to note that A.cau_ornamental clusters close to A.cau_Bolivia_PI642741. Suvarna, an accession/landrace from India, often classified as *A. hypochondriacus* in the literature, clearly clusters with *A. cruentus and* shows high similarity to the accession A.cru_Mexico_PI477913, also classified as *A. cruentus*. This is also obvious from the stem solidness as reported by Malligawad and Patil (2010) and as shown in Figure 2E.

**FIGURE 4:**
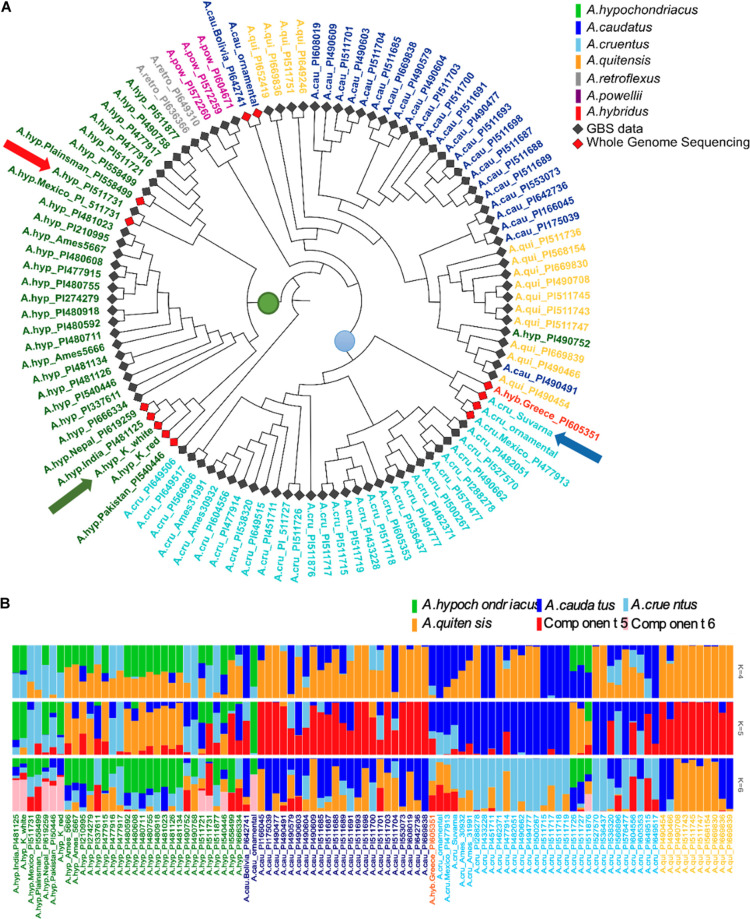
**(A)** Shows a classification of 94 accessions from GBS data and 13 from WGS data after normalization of the two sequencing approaches using 271,305 SNPs. The red arrow indicates the position of A.hyp_Plainsman_PI558499 WGS sample next to its corresponding GBS, green arrow indicates position of A.hyp_K_white and blue arrow indicates the position of A.cru_Suvarna in the phylogenetic tree. **(B)** Genetic admixture analysis of *A. hypochondriacus, A. caudatus, A. cruentus*, and *A. quitensis.*

In Figure 4A, an attempt was made to decorate the classification of 94 accessions generated both using GBS data with WGS data for landraces generated here. Since GBS only covers 10% of the genome, there is a need to normalize the variants from WGS data for comparison. One way to do this would be to identify and use the alleles found from GBS data with the respective alleles found from WGS. However, this produced skewed classification because of variation in the depth of sequencing between GBS and WGS while calling variants. We have devised a method to normalize for the same during the variant calling (see section “Materials and Methods”).

As a second approach, the phylogenetic tree shown in Figure 4A and generated using AhKP as reference (Figure 4A) combines variants called for the 94 accessions using both raw genotyping-by-sequencing (GBS) data from public sources (Wu and Blair, 2017) and whole-genome sequencing (WGS) data for listed accessions in Supplementary Table S2. The normalization is validated by clustering of both WGS and GBS data from A.hyp_Plainsman_PI558499 and A.hyp_Mexico_PI511731 close to each other (Figure 4A, red arrow). The tree in Figure 4A is very similar to that reported in Wu and Blair (2017) with 10,688 SNPs from accessions using GBS technology. However, according to our classification with 271,305 SNPs, by combining GBS and WGS data, we reproduce the observation made by Wu and Blair (2017). The taxonomy-based classification seems reproducible with green being *A. hypochondriacus*, light blue being *A. cruentus*, dark blue being *A. caudatus* and yellow being *Amaranthus quitensis*. Our reclassification of A.hyp_PI490752 as *A. quitensis*, which was originally annotated as *A. hypochondriacus*, seconds the observation made by Wu and Blair (2017). However, we report a new observation that PI649506 is a *A. hypochondriacus* originally annotated as *A. cruentus*. Interestingly, similar to Figure 2, A.hyp_K_white and A.hyp_K_red, both landraces from India cluster closely together along with accessions A.hyp_Nepal_PI619259 and A.hyp_India_PI481125. A.cru_Suvarna, yet another landrace sequenced and reported here is clearly classified as *A. cruentus*. This was supported by the solid stem characteristics of *A. cruentus* for A.cru_Suvarna as reported by Malligawad and Patil (2010). Besides, the seed sizes shown in Figure 2F also validate classification for Suvarna as *A. cruentus* with relatively bigger seed size.

ADMIXTURE analysis shown in Figure 4B suggests that there is significant gene flow between *A. caudatus* and *A. quitensis*. At *K* = 4 and 5 there is no resolution between species except *A. hypochondriacus*. However, at *K* = 6 there is resolution in components for all four species with green for *A. hypochondriacus*, dark blue for *A. cruentus* and major yellow representing components of *A. caudatus* and *A. quitensis*. At *K* = 6, we also see pink components uniformly present in all the *A. hypochondriacus* from the Indian subcontinent with the exception of accessions A.hyp_Mexico_PI511721(GBS), A.hyp_Mexico_PI511731(GBS). Interestingly, A.hyp_Mexico_ PI511721 clusters with A.hyp_Plainsman_PI558499, which does not show any pink components.

### Development of Tissue-Specific Gene Expression Atlas of Amaranth

**FIGURE 5:**
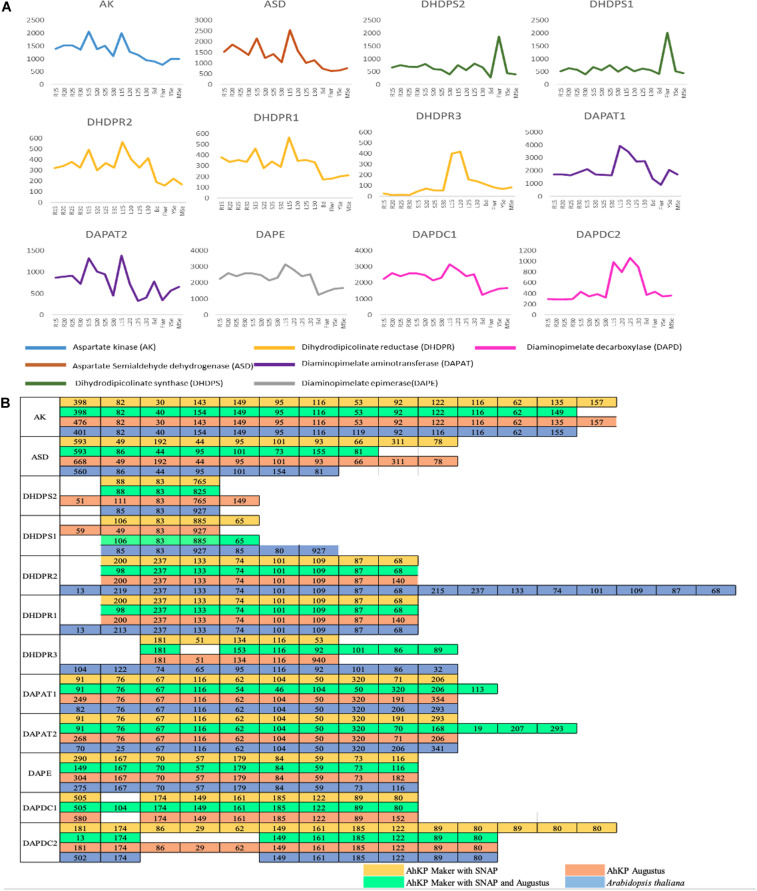
**(A)** Gene expression profile of 12 genes involved in lysine biosynthesis pathway across different developmental stages (15, 20, 25, and 30 days) of different tissues (Rt, root; St, stem; L, leaf; Bud, Flwr, Flower; YSe, young seed; and MSe, mature seed). **(B)** Comparison of CDS sizes (nucleotides) of lysine biosynthesis pathway genes predicted in AhKP with *Arabidopsis.*

In a previous report, our lab had sequenced and reported developmental transcriptome of A.hyp_K_white from several tissues (Sunil et al., 2014, 2017). Here, to understand/translate the high-lysine phenotype and to validate the gene structure obtained from our annotation efforts, the transcriptomes have been mapped to AhKP reference and the expression profiles of the predicted genes have been generated across the developmental stages. The bam files of each sample can be visualized in the respective genome browser (link to the same is available in the data availability section) Also, the expression profiles of all the 12 predicted genes from the lysine pathway across developmental stages is provided in Figure 5A along with the corresponding exon number and sizes compared to *Arabidopsis* Figure 5B. Also, the browser can be queried using the accessions of *Arabidopsis* to visualize the expression profile of the corresponding orthologs on AhKP.

## Discussion

Here, a chromosome level assembly (AhKP) of a landrace, A.hyp_K_white, under contiguous cultivation in India for over several centuries is reported. A draft genome for the same landrace was reported by our group in 2014 (Sunil et al., 2014). The assembly reported here is obtained using relatively low coverage of long reads from PacBio RSII technologies in conjunction with a high-quality reference for another distal accession of the same species A.hyp_Plainsman_PI558499. We used multiple assembly tools adapted/developed recently to handle error-prone long reads and merged these assemblies with the contigs from our previously reported draft assembly. The assembly statistics of the initial assembly was sufficient for reference-based scaffolding using both the simulated mate-pairs from the reference genome and raw HiC data for Plainsman from public resources (Lightfoot et al., 2017). RepeatMasker analysis classified 50.5% (196421031 bp) of the AhKP genome as repetitive sequences. Annotation using the MAKER (Campbell et al., 2014) annotation pipeline predicted 18,858 gene models which has been validated for the 12 genes from lysine biosynthesis pathway by comparing it to *Arabidopsis* gene model as shown in Figure 5.

Whole-genome sequencing from a few landraces and ornamental varieties generated in-house and several other accessions from public sources are clustered using 20,548 SNPs out of the 27,658 SNPs reported for grain amaranths (Figure 3A). Figure 3 shows classification using both the 20,548 out of 27,658 reported SNPs covered in all samples (Figure 3A) and ∼6 million variants called from mapping WGS reads to AhKP and A.hyp.V2.1 reference, respectively, (Figures 3B,C). In that, the genome of the landrace AhKP, reported here, is closest to accession A.hyp_Nepal_PI619259 and to A.hyp_India_PI481125. However, A.hyp_Plainsman_PI599488 clusters in a distal clade with A.hyp_Mexico_PI511731 in both Figures 3B, 4A as expected because Plainsman is a cross between *A. hypochondriacus* from Mexico and *A. hybridus* from Pakistan (Guillen-Portal et al., 1999). Interestingly, the only *A. hybridus* accession from Greece (A.hyb_Greece_PI605351) included in the classification is not in the same clade as Plainsman but, instead, clusters along with all accessions from *A. cruentus* (near blue arrow in Figure 4A). Also, the two landraces under *A. hypochondriacus*, A.hyp_K_white and A.hyp_K_red, cluster apart within the same clade validating our observation that the seeds of these two varieties faithfully produce plants with inflorescence unique to the respective phenotype. Besides, a C0t analysis shown in Supplementary Figure S2 suggests distinct dissociation time for simple repeat between these two accessions.

The successful integration of WGS and GBS data attempted here, is apparent from the clustering of variants from WGS and GBS data for the same accession together as marked with red arrow in Figure 4A. Figure 4 validates taxonomy-based classification (color-coded accessions) of the majority of the accessions and landraces. However, a few accessions are now reclassified. The accession PI490752 originally classified as *A. hypochondriacus* now classifies under *A. quitensis*. *A. hybridus* with accession of PI605351 clusters in the same clade as Suvarna with another accessions (PI477913) from *A. cruentus*. All the accessions from *A. quitensis* and *A. caudatus* clusters together in a single clade with two branches of *A. quitensis* enclosing *A. caudatus*, which is also reported using only GBS data using A.hyp.V.2.1 as reference (Lightfoot et al., 2017). This suggests that *A. caudatus* is a major clade under *A. quitensis*. ADMIXTURE analysis shown in Figure 4B, also suggests that there is significant gene flow between *A. caudatus* and *A. quitensis*. At *K* = 4, a significant component of *A. quitensis* is found in all four species. However, at *K* = 5 and 6 components of *A. quitensis* is profound only in *A. caudatus*. At *K* = 6 other unique components within *A. hypochondriacus* gets resolved. For example, there is a component (Figure 4B, pink) only present in all *A. hypochondriacus* from India, which is missing in Plainsman.

Suvarna (R 104-1 -1), a pureline released by University of Agricultural Sciences (UAS), Bangalore-1992 from the material “Rodale Plus” received from Rodale Institute (Rathod, 2017) has previously been classified as *A. hypochondriacus* in Meera et al., 2014. In this report, Suvarna is undoubtedly classified as *A. cruentus* based on 20,548 reported SNPs and roughly 6 million variants covered in WGS data (Figure 3). Also, morphological features like stem solidness and total height at maturity as mentioned by Malligawad and Patil (2010) and seed size (Figure 2F) supports this classification.

We hypothesize that the only component showing light blue that is common between Suvarna and A.hyp_K_white in the ADMIXTURE with *K* = 5 and 6 (Figure 3B) holds the genotype responsible for inflorescence within this haplo-block.

We believe that this is the first demonstration of generating a cost-effective *de novo* assembly for a landrace utilizing low coverage PacBio reads in conjunction with the genome and HiC data from another accession. Since this landrace is more closely similar to all other landraces and accessions for *A. hypochondriacus* from India and South Asia (Supplementary Table S1), AhKP offers a better reference for the improvement of grain amaranth crops in South Asia. The landrace A.hyp_K_white is currently being used to identify mutations in targeted loci for a given desirable phenotype from a germplasm collection using eco-TILLING and to discover novel mutations that result in desirable traits like determinate growth, enhanced seed yield, seed lysine content and oil content using TILLING-based approaches.

## Materials and Methods

### Samples

Seeds of A.hyp_K_white, A.hyp_K_red were obtained from local market in Karnataka, India, A.cru_ornamental, A.cau_ornamental from Park seeds and A.cru_Suvarna from Gandhi Krishi Vigyana Kendra (GKVK), Bengaluru, Karnataka, India.

### Source of Data Used in This Work

Plainsman reference: Phytozome^1^ *A. hypochondriacus* genome V.2.1(A.hyp.V.2.1) (Lightfoot et al., 2017) GBS: Blair et al. Front Plant Sci. 2017 (Wu and Blair, 2017) WGS (Lightfoot et al., 2017)^2^.

### Isolation of Genomic DNA

Amaranth A.hyp_K_white, A.hyp_K_red, A.cru_ornamental, A.cau_ornamental, and A.cru_Suvarna variety were grown at IBAB (Figure 2). Genomic DNA was extracted from fresh leaves using the DNeasy Mini Plant DNA Extraction kit (Qiagen) following the manufacturer’s protocol and quantified using fluorometry (Qubit 2.0, Invitrogen).

### Library Preparation and Sequencing

Library preparation was done for Illumina whole genome sequencing in-house and outsourced for PacBio RSII sequencing.

Whole Genome libraries for A.hyp_K_white, A.hyp_K_red, A.cru_ornamental, A.cau_ornamental and A.cru_Suvarna were prepared using the TruSeq DNA Sample Preparation Kit (Illumina) by following the manufacturer’s low throughput protocol. One microgram and 10 μg of the DNA were used for the preparation of Paired-End (PE) and Mate-Pair (MP) libraries, respectively. DNA was sheared using Adaptive Focused Acoustic technology (Covaris, Inc.) to generate fragments of desired insert size. The average insert size was around 200 bp for PE libraries and 1.75, 3, 5, and 10 kb for four MP libraries.

Briefly, for PE libraries, the fragmented DNA was end-repaired, 3′-adenylated, ligated with Illumina adapters, and PCR enriched with Illumina sequencing indexes. For MP libraries, the fragmented DNA was end-repaired, followed by end labeling using the biotin-dNTP mix, size selected and later, circularized using circularization ligase. The circular DNA was sheared again as explained earlier, and the biotinylated fragments were purified using streptavidin beads (Dynabeads^TM^ M-280 Streptavidin, Invitrogen), the fragments were end-repaired, 3′-adenylated and ligated with Illumina adapters. Further, the biotinylated, adapter-ligated immobilized DNA were enriched by PCR. The size selection for all the libraries were done using solid-phase reversible immobilization (SPRI) beads (Agencourt AMPure XP Beads) from Beckman Coulter. The quality, quantity, and size distribution of the libraries were evaluated using Qubit (Invitrogen) and TapeStation (Agilent) (Sunil et al., 2014). The clusters were generated in cBot and paired-end sequenced on Illumina HiSeq 2500 platform.

Whole-genome PacBio sequencing was done by Molecular Biology and Genomic Core, Washington University using P5/C3 chemistry on the Pacific Biosciences RSII platform. This platform is a single-molecule, real-time (SMRT) sequencing machine that uses a sequencing-by-synthesis method to generate good quality very long reads.

#### Assembling the Raw Data

**FIGURE 6:**
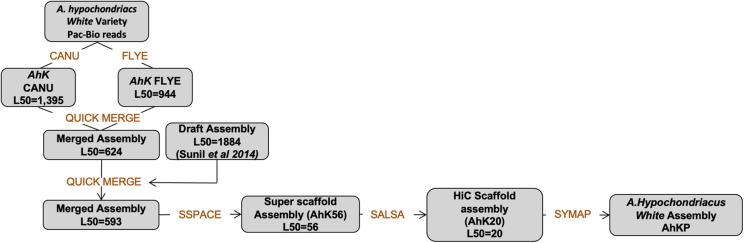
Workflow used in the assembly of AhKP.

The raw PacBio data was assembled using Canu (Koren et al., 2017) and Flye (Kolmogorov et al., 2019) independently. The two assemblies obtained were then merged together using Quickmerge (Chakraborty et al., 2016). This was further improved by merging the Illumina assembly from the draft genome reported elsewhere and polished using the Illumina reads. The scaffolds from this step are further improved with simulated mate pairs using wgsim (Li, 2020) from Plainsman with SSPACE. At this stage, the scaffolds were long enough to allow the use of HiC data generated for Plainsman to obtain high-resolution assembly (AhK20). Further, we generated synteny of the AhK20 against A.hyp.V.2.1 using Symap (Soderlund et al., 2011) based on which AhK20 was improved to the final AhKP assembly. The flowchart below (Figure 6) shows the pipeline used to obtain the final assembly.

### SNP Analysis and Construction of Phylogenetic Tree for Whole Genome Samples

The Illumina data of all the plants with accessions listed in Supplementary Table S2 were downloaded from NCBI SRA (SRP061623). The public and in house generated data were mapped to A.hyp.V.2.1 and AhKP reference using bowtie2 (Langmead and Salzberg, 2012). From the mapped reads, variants were called using samtools (v1.9) mpileup (Li et al., 2009) and bcftools (v1.9) (Li et al., 2009). The variants were filtered using bcftools (Li et al., 2009) with the criteria of QUAL (quality) greater than 10 and DP (read depth) greater than 3 and INDELs were also removed. The files were then merged, and the genotype matrix was created using a custom script. Further, the regions covered in sequencing were identified using bedtools genomecov (Quinlan and Hall, 2010) from bam files, and the regions, which were not covered in sequencing in any of the samples, were removed from the genotype matrix. The phylogenetic tree was constructed from the genotype matrix using the clustering algorithm hclust and SNPRelate under R and Bioconductor package (Zheng et al., 2012).

For the 27,658 SNP positions 150 base pair sequences were downloaded from public sources and coordinates for all 27,658 positions on A.hyp.V.2.1 were extracted by BLAST alignment. A separate VCF file was made for all the 13 datasets as listed in Supplementary Table S2 with the respective alleles at these positions. Only 20,548 SNPs were commonly covered in all 13 WGS datasets and were used during classification. The resultant VCF files were merged and based on the presence or absence of SNPs, a binary matrix was constructed from which a phylogenetic tree was obtained as mentioned above.

### Classification Using GBS and WGS Data

GBS raw data of 95 accessions were downloaded from Wu and Blair (2017) of which *A. palmeri* was excluded from the analysis because of the reported high level of missing data. The reads were demultiplexed using GBSX (Herten et al., 2015) using the provided barcode sequences. Post demultiplexing, the reads were mapped to AhKP using bowtie2 (Langmead and Salzberg, 2012) and SNP calling was done using the method described in the above section.

**FIGURE 7:**
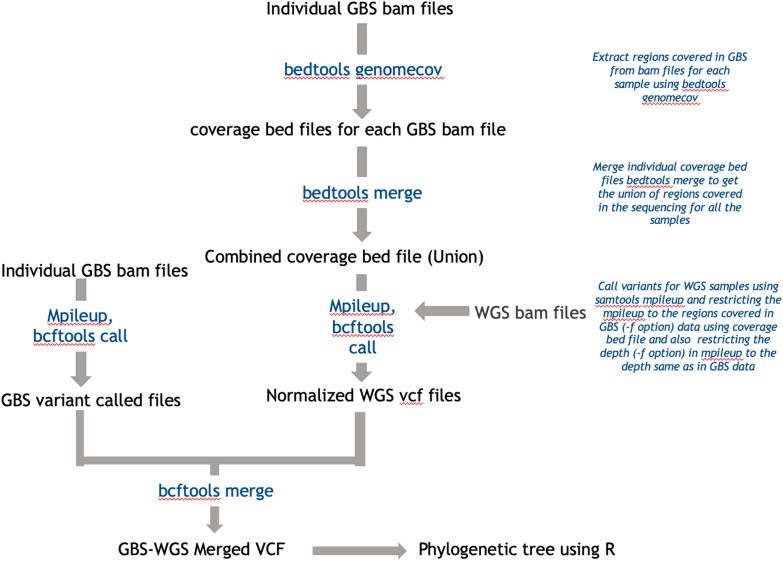
Workflow used in integrating WGS data with GBS.

To combine WGS data and GBS data, we created GBS like data from whole-genome reads. For this, the regions covered in GBS data were extracted using bedtools genomecov (Quinlan and Hall, 2010) for all the accessions, and the regions covered were merged to get a maximum possible region covered in GBS sequencing for all the accessions combined. These regions were used to restrict the variant calling from whole-genome data to only the regions covered in GBS. Also, the read depth considered during variant calling was restricted using samtools mpileup to 10 to match the depth of GBS data (Li et al., 2009). The SNPs were merged and used for phylogenetic classification. A detailed summary of this method of normalization has been explained in Figure 7. Also, the tree generated prior to the normalization method has been presented in Supplementary Figure S3 for highlighting the extent of improvement upon normalization.

#### Admixture Analysis

Population genetic diversity was analyzed for four Amaranth species (*A. hypochondriacus, A. caudatus, A. cruentus, and A. quitensis).* Only 97 out of 107 samples from both GBS and WGS data were filtered based on their good clustering and bigger sample size. The merged SNP file was processed using PLINK (Purcell et al., 2007) and ADMIXTURE (v1.3) was used to analyze the population structure (Alexander and Lange, 2011). We then selected *K* =4–6 as the optimal number subpopulation “K” based on the lowest cross-validation (c.v) error value plot as reported by ADMIXTURE(v1.3) where we see a dip in the c.v value at *K* = 4 and 6 (Supplementary Figure S4).

#### Genome Annotation and Repeat Analysis

Repeat elements for the Plainsman and the A.hyp_K_white variety of *A. hypochondriacus* assemblies were predicted using RepeatModeler version 2.0.1 (Flynn et al., 2019) along with LTR discovery. The two predicted libraries of repeat elements were merged together and repeat masking was done using RepeatMasker version 4.1.0 (Smit and Hubley, 2008–2015).

Annotation of AhKP was done using multiple approaches (i) Augustus (Hoff and Stanke, 2019) (v3.2.3) prediction using *Arabidopsis* as model and (ii) MAKER (Campbell et al., 2014) genome annotation pipeline with (with and without Augustus) default parameters, was used for AhKP annotation. Maker pipeline includes *de novo* assembled amaranth transcriptome with 125581 scaffolds, repeat elements predicted by RepeatModeler and *Arabidopsis* proteome (TAIR10) (Berardini et al., 2015). SNAP (Korf, 2004) and Augustus were also used to predict gene models and used in the subsequent rounds of MAKER (Campbell et al., 2014).

Genes involved in lysine biosynthesis pathway were identified by BLASTP (Altschul et al., 1990) analysis using *Arabidopsis* proteins.

#### Transcriptome Analysis

Raw transcriptome reads generated in our earlier work and reported in Sunil et al. (2017) from 16 developmental stages including leave, stem and root from 15,20,25 and 30 day plant, bud, flower, young seed and mature seed were mapped to AhKP reference using bowtie2 (Langmead and Salzberg, 2012). The mapped files were processed using samtools (Li et al., 2009) and raw read count was counted for all predicted genes using bedtools multiBamCov (Quinlan and Hall, 2010). Further DESeq2 (Love et al., 2014) was used to get normalized read counts of all the predicted genes using the MAKER (Campbell et al., 2014) gene annotation pipeline.

### Genome Browser and Database

The Amaranth database is running on EC2 instances of Amazon cloud service (AWS). The database is built using HTML5, bootstrap and JavaScript. The database consists of a landing page, genome browser and NCBI BLAST (Altschul et al., 1990) tool. This database is made from a framework provided by Meghagen LLC. JBrowse (Skinner et al., 2009) is JavaScript and html based genome browser provides the solution for visualization of various kinds of genomic data such as FASTA, BAM, GFF, VCF, and bigwig etc., Data for downloading and JBrowse is stored on the cloud and made available for research purposes. The menus on the database page will redirect you to the download as well as tool page. Users can access the Jbrowse by clicking on the Genome browser button or using the tools menu. Users can access the database from the link given in the data availability section. The database is also integrated with graphical visualization for gene expression data of 16 developmental stages with query search options.

## Data Availability Statement

This Whole Genome Shotgun project has been deposited at DDBJ/ENA/GenBank under the accession JPXE00000000. The version described in this paper is version JPXE02000000.

## Author Contributions

SPD: classification, characterization and writing of the manuscript. SJ: assembly of AhKP. SR: library preparation and aiding writing of manuscript. KRR: assembly and analysis of other landraces. SW: development of genome browser. NH: transcriptome analysis. SD: DNA isolation and repeat analysis. MS: PacBio data, developmental transcriptome and taxonomic classification of landraces. ER: forinitiating translational work and validating transcripts. BC: for overseeing the experimental component of the project. PGS: for guidance throughout the project. SS: for overseeing the project and writing of the manuscript.

## Conflict of Interest

The authors declare that the research was conducted in the absence of any commercial or financial relationships that could be construed as a potential conflict of interest.

## Footnotes

1 http://phytozome.jgi.doe.gov/

2 https://www.ncbi.nlm.nih.gov/sra?term=SRP061623
