# Supplementary material for: Classification of Grain Amaranths Using Chromosome-Level Genome Assembly of Ramdana, A. hypochondriacus
Source: Front Plant Sci. 2020 Nov 11;11:579529. doi: 10.3389/fpls.2020.579529 (PMC7686145; doi:10.3389/fpls.2020.579529)
Supplement: Supplementary file 1 [file Data_Sheet_1.docx]

**SUPPLEMENTARY DATA**

Supplementary Figure S1 - *A. hypochondriacus* Plainsman PI558499. Image from <https://www.southernexposure.com/products/amaranth-plainsman/>


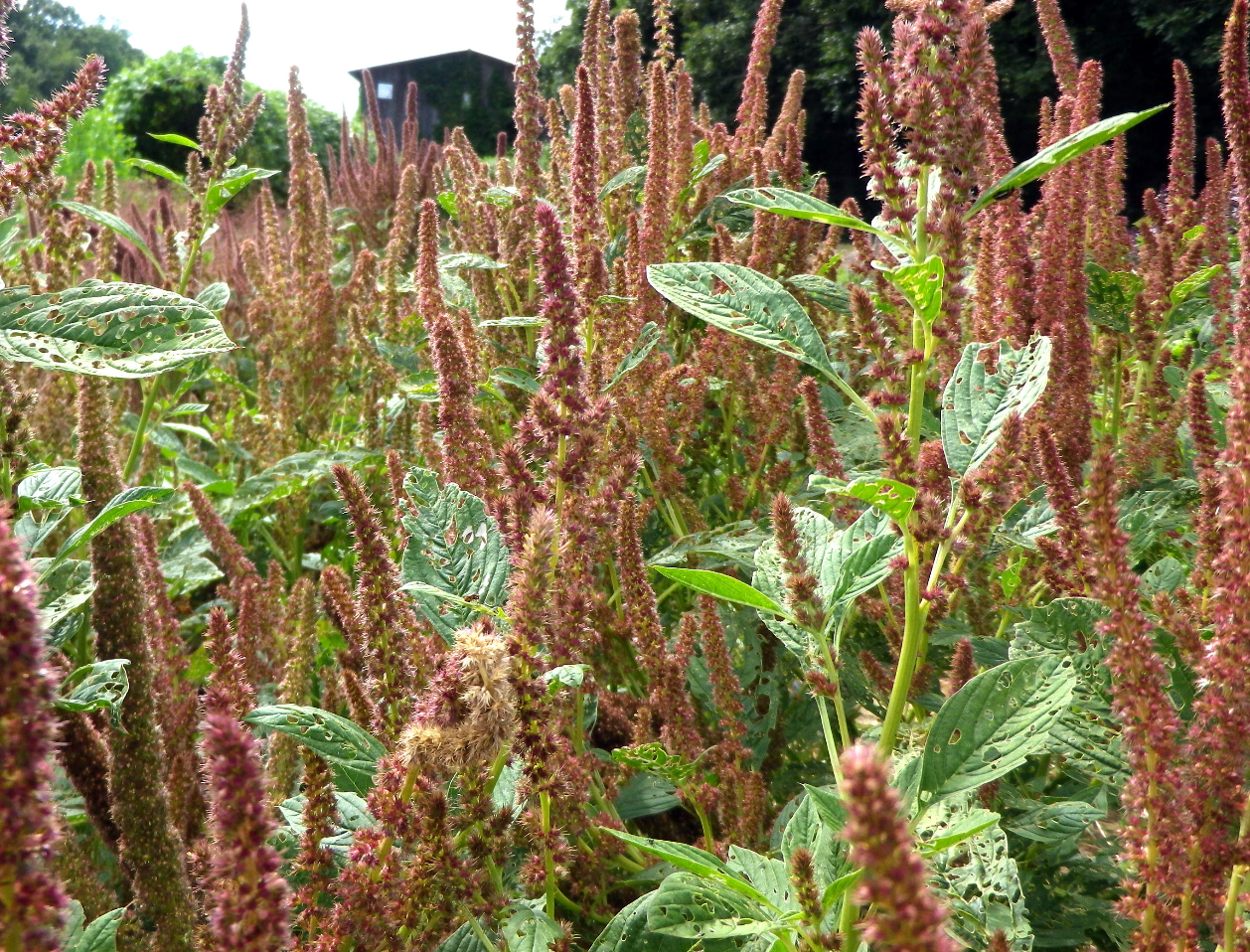


Supplementary Figure S2 - C_0_t analysis of A.hyp_K_white and A.hyp_K_red

**
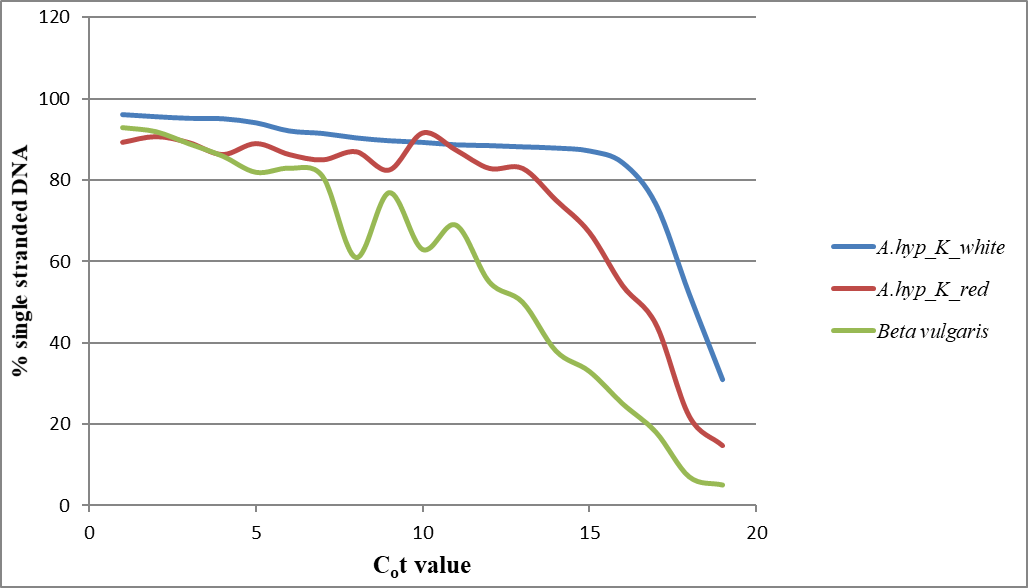
**


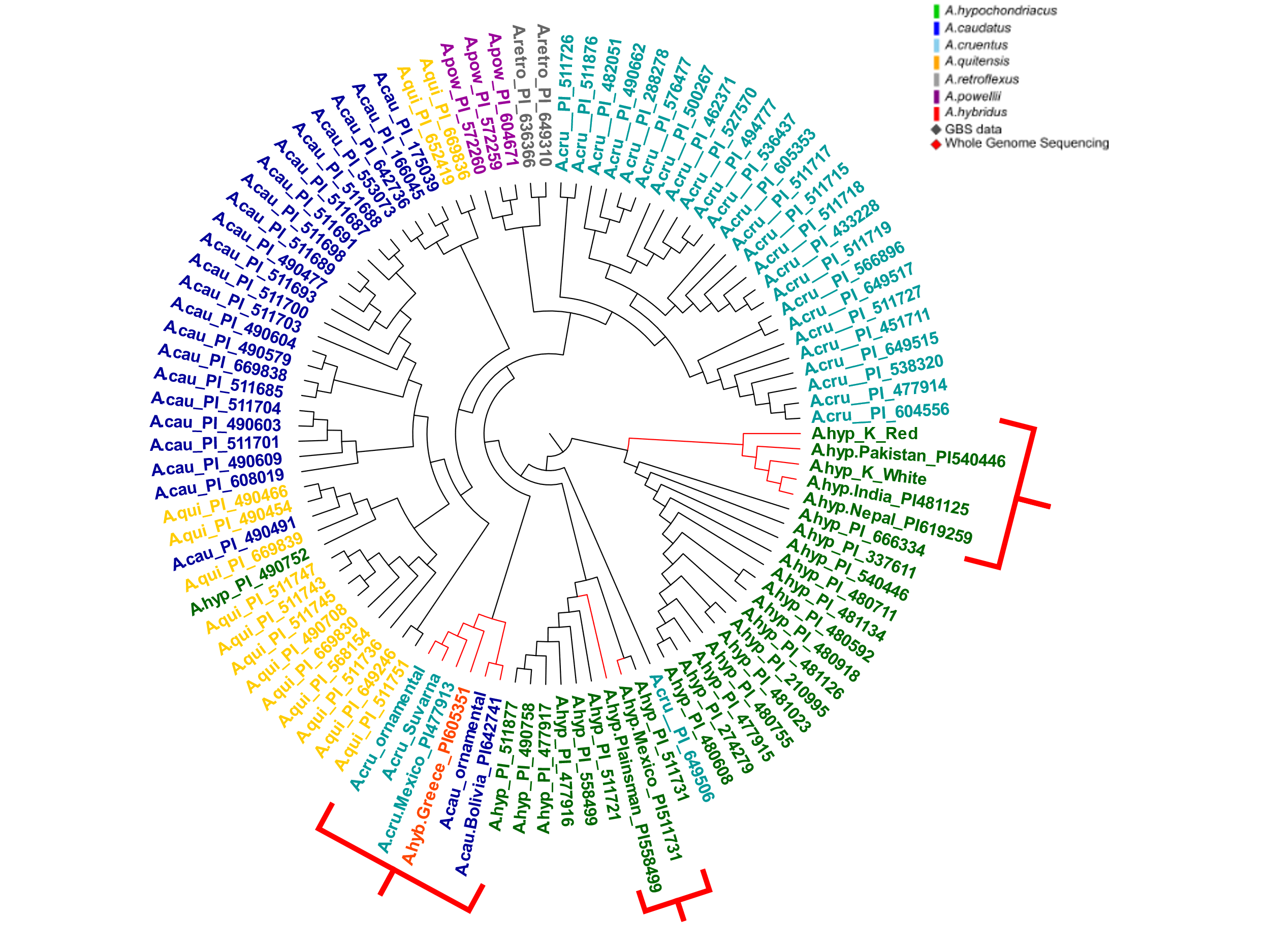
Supplementary Figure S3- Tree generated using non-normalized WGS-GBS data.


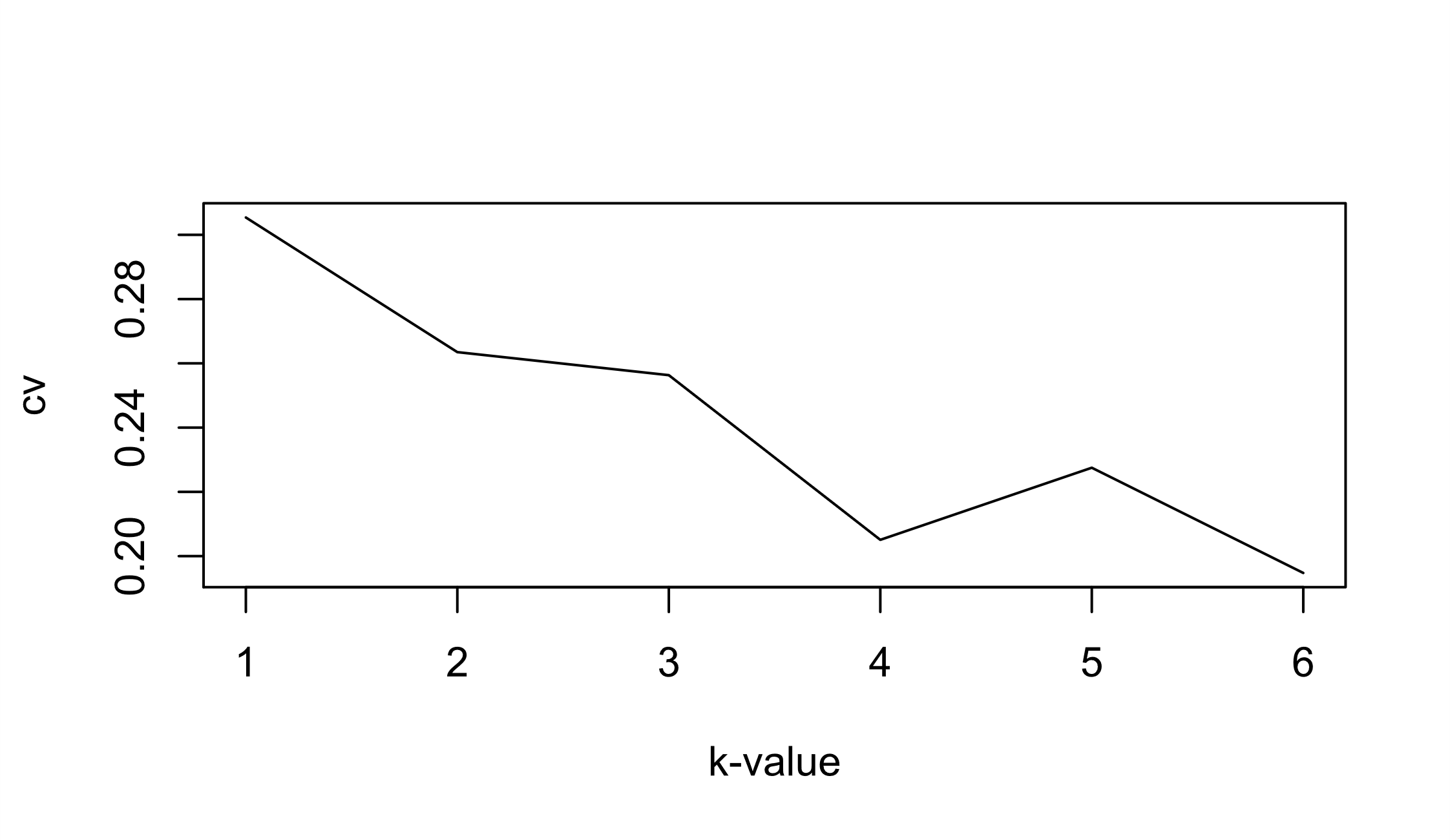


Supplementary Figure S4- Cross validation error value plot for different K values of admixture analysis.

Supplementary Table S1- Details of in-house sequenced plants

|  | **Total no of reads** | **Coverage** |
| --- | --- | --- |
| **A.hyp_K_white** | 469749648 | 87.3357055 |
| **A.cau_ornamental** | 266702040 | 53.1278964 |
| **A.cru_ornamental** | 353363298 | 69.2869212 |
| **A.hyp_K_red** | 447933760 | 111.03960 |
| **A.cru_Suvarna** | 501687774 | 147.555228 |

Supplementary Table S2- SNP count of all whole genome samples on both references

| **Name** | **Alias** | **On A.hyp.V.2.1** | **On AhKP** |
| --- | --- | --- | --- |
| *A. caudatus* (Bolivia) PI 642741 | A.cau_PI642741 | 2846014 | 2424857 |
| *A. cruentus* (Mexico) PI 477913 | A.cru_PI477913 | 3263942 | 2773417 |
| *A. hypochondriacus* (India) PI481125 | A.hyp_PI481125 | 886235 | 117514 |
| *A. hypochondriacus* K white (India) | A.hyp_K_white | 901042 | 110024 |
| *A. hypochondriacus* Plainsman PI558499 | A.hyp_PI558499 | 78926 | 765147 |
| *A. hypochondriacus* (Nepal) PI619259 | A.hyp_PI619259 | 881424 | 112943 |
| *A. hypochondriacus* (Pakistan)PI540446 | A.hyp_PI540446 | 937366 | 196203 |
| *A. hypochondriacus* K red (India) | A.hyp_K_red | 1006061 | 193013 |
| *A. hypochondriacus* (Mexico) PI511731 | A.hyp_PI511731 | 1248769 | 738991 |
| Suvarna (India) | A.cru_Suvarna | 3267631 | 2796817 |
| *A. hybridus* (Greece) PI605351 | A.hyb_PI605351 | 3280993 | 2783846 |
| *A. caudatus* (Love-Lies-Bleeding) | A.cau_ornamental | 2954432 | 2138832 |
| *A. cruentus* (Autumn Touch) | A.cru_ornamental | 3428292 | 2920539 |

Supplementary Table S3- Agronomic trait comparison between A.hyp_K_white and A.hyp_Plainsman_PI558499

|  | A.hyp_K_white ~PI619259 | Plainsman PI558499 (Baltensperger 1999) |
| --- | --- | --- |
| Seed color | Light | Light |
| Inflorescence | White | White/Red |
| Height | 5-6 feet | 4 feet |
| Seed length/width | ~450/450 µm (See Figure 2.f) | NA |
| Yeild | 3-4g per plant | 2-3g per plant |
